# Genetic Deficiency of Glycogen Synthase Kinase-3β Corrects Diabetes in Mouse Models of Insulin Resistance 

**DOI:** 10.1371/journal.pbio.0060037

**Published:** 2008-02-19

**Authors:** Katsuya Tanabe, Zhonghao Liu, Satish Patel, Bradley W Doble, Lin Li, Corentin Cras-Méneur, Sara C Martinez, Cris M Welling, Morris F White, Ernesto Bernal-Mizrachi, James R Woodgett, M. Alan Permutt

**Affiliations:** 1 Division of Endocrinology, Metabolism, and Lipid Research, Washington University School of Medicine, St. Louis, Missouri, United States of America; 2 Samuel Lunenfeld Research Institute, Mount Sinai Hospital, Toronto, Ontario, Canada; 3 Howard Hughes Medical Institute, Children's Hospital, Harvard Medical School, Boston, Massachusetts, United States of America; University of Cambridge, United Kingdom

## Abstract

Despite treatment with agents that enhance β-cell function and insulin action, reduction in β-cell mass is relentless in patients with insulin resistance and type 2 diabetes mellitus. Insulin resistance is characterized by impaired signaling through the insulin/insulin receptor/insulin receptor substrate/PI-3K/Akt pathway, leading to elevation of negatively regulated substrates such as glycogen synthase kinase-3β (Gsk-3β). When elevated, this enzyme has antiproliferative and proapoptotic properties. In these studies, we designed experiments to determine the contribution of Gsk-3β to regulation of β-cell mass in two mouse models of insulin resistance. Mice lacking one allele of the insulin receptor (*Ir^+/−^*) exhibit insulin resistance and a doubling of β-cell mass. Crossing these mice with those having haploinsufficiency for Gsk-3β (*Gsk-3β^+/−^*) reduced insulin resistance by augmenting whole-body glucose disposal, and significantly reduced β-cell mass. In the second model, mice missing two alleles of the insulin receptor substrate 2 (*Irs2^−/−^*), like the *Ir^+/−^* mice, are insulin resistant, but develop profound β-cell loss, resulting in early diabetes. We found that islets from these mice had a 4-fold elevation of Gsk-3β activity associated with a marked reduction of β-cell proliferation and increased apoptosis. *Irs2^−/−^* mice crossed with *Gsk-3β^+/−^* mice preserved β-cell mass by reversing the negative effects on proliferation and apoptosis, preventing onset of diabetes. Previous studies had shown that islets of *Irs2^−/−^* mice had increased cyclin-dependent kinase inhibitor p27^kip1^ that was limiting for β-cell replication, and reduced Pdx1 levels associated with increased cell death. Preservation of β-cell mass in *Gsk-3β^+/−^Irs2^−/−^* mice was accompanied by suppressed p27^kip1^ levels and increased Pdx1 levels. To separate peripheral versus β-cell–specific effects of reduction of Gsk3β activity on preservation of β-cell mass, mice homozygous for a floxed *Gsk-3β* allele (*Gsk-3^F/F^*) were then crossed with *rat insulin promoter-Cre* (*RIP-Cre*) mice to produce β-cell–specific knockout of Gsk-3β (*βGsk-3β^−/−^*). Like *Gsk-3β^+/−^* mice, *βGsk-3β^−/−^* mice also prevented the diabetes of the *Irs2^−/−^* mice. The results of these studies now define a new, negatively regulated substrate of the insulin signaling pathway specifically within β-cells that when elevated, can impair replication and increase apoptosis, resulting in loss of β-cells and diabetes. These results thus form the rationale for developing agents to inhibit this enzyme in obese insulin-resistant individuals to preserve β-cells and prevent diabetes onset.

## Author Summary

Diabetes is often characterized by a failure of insulin production by pancreatic β-cells to properly regulate glucose homeostasis. Insulin resistance can lead to β-cell failure, and our studies have focused on elucidating the mechanisms involved in this postnatal failure. In this study, we evaluated a new, negatively regulated enzyme of the insulin signaling pathway, glycogen synthase kinase 3 (Gsk-3), specifically within insulin-producing pancreatic β-cells. When this enzyme is elevated, it can impair replication and increase cell death, resulting in loss of insulin-producing cells and diabetes. Gsk-3 is also known to regulate cell death and proliferation in neurons. We assessed the role of Gsk-3 on glucose homeostasis in two different mouse models of insulin resistance. We demonstrated that genetically reducing the levels of Gsk-3β in the insulin-resistant mouse improved glucose homeostasis. In another model in which severe insulin resistance is associated with destruction of β-cells, reducing Gsk-3β not only preserved β-cells by increasing proliferation and reducing cell death, but it also corrected diabetes. Controlling activity of Gsk-3 could lead to new hopes for maintaining or improving β-cell number and prevention of diabetes.

## Abstract 3

The insulin signaling pathway negatively regulates glycogen synthase kinase 3. The current studies demonstrate that this enzyme is rate limiting for pancreatic β-cell replication and survival.

## Introduction

Despite treatment with agents that enhance β-cell function and insulin action, reduction in β-cell mass is relentless in type 2 diabetes (T2DM) [1–4]. Why β-cells fail in some individuals is a central issue in diabetes research today. The molecular mechanisms enabling β-cell adaptation to insulin resistance are being discovered primarily in animal models [5–7]. Important genetic models have focused on the requirement for insulin signaling through β-cell insulin/insulin-like growth factor 1 (IGF1) receptors (reviewed in [8,9]). Whereas mice with total-body deficiency for insulin receptor substrate 1 (*Irs1^−/−^*) have insulin resistance and significant expansion of β-cell mass, insulin receptor substrate 2–deficient mice (*Irs2^−/−^)* have insulin resistance yet develop postnatal β-cell loss and severe diabetes (reviewed in [10]). In this model and in others, the primacy of PI-3K/Akt activity in expansion and postnatal maintenance of β-cell mass was apparent (reviewed in [11,12]). The remarkable ability of β-cell mass to expand via enhanced proliferation and reduced apoptosis was demonstrated in transgenic mice expressing constitutively active Akt in β-cells [13,14], illustrating the potential importance of this pathway for expanding β-cells in patients and perhaps resisting the apoptosis that accompanies long-standing diabetes. Knowing that increased expression of Akt in β-cells leads to marked expansion, these results have focused interest on the role of two negatively regulated Akt substrates, FoxO1 and Gsk-3β, each known to regulate carbohydrate and lipid metabolism in insulin target tissues while also exhibiting antiproliferative and proapoptotic properties when expressed at high levels [15,16]. There is substantial evidence, mostly from overexpression of a constitutively nuclear FoxO1, that FoxO1 has detrimental effects on β-cell proliferation and survival [17]. On the other hand, there is little known about the effects of expression of Gsk-3β on β-cell proliferation and/or survival.

Glycogen synthase kinase-3 (Gsk-3) was originally identified as a serine/threonine kinase that inactivates glycogen synthase [18]. Early studies showed that insulin inhibits Gsk-3 activity through PI-3K/Akt-induced phosphorylation promoting glycogen synthesis and glucose disposal [19–22]. Later, the enzyme was shown to affect many cellular processes, including transcription, translation, cell cycle regulation, and apoptosis [16,23–27]. Mammals express two isoforms, Gsk-3α and Gsk-3β, which share similar kinase domains but differ considerably in their termini. Inactivation of Gsk-3β appears to be the major route by which insulin activates glycogen synthesis [22,28], and recent studies have demonstrated that elimination of Gsk-3β is more effective at promoting neuronal survival than is elimination of Gsk-3α [29].

Gsk-3 activity has been shown to be increased in peripheral tissues in diabetic animals and patients [30–32], and diabetes was reversed in obese diabetic mice treated with Gsk-3 inhibitors [33–35]. Because inhibitors have differing degrees of kinase specificity, Gsk-3–deficient genetic models were created. Disruption of the Gsk-3β gene in mice results in embryonic lethality [23], yet mice with loss of one allele (*Gsk-3β^+/−^*) are viable and express reduced levels of protein and enzymatic activity [23]. Although *Gsk-3β^+/−^* mice have been little studied, they have been shown to have behavioral effects similar to lithium-treated mice, suggesting that Gsk-3β is the main determinant of Gsk-3 activity in the nervous system [36]. The availability of these *Gsk-3β^+/−^* mice provided the opportunity to assess the role of Gsk-3β on insulin sensitivity and pancreatic β-cell function.

Mice haploinsufficient for the insulin receptor (*Ir^+/−^*) have insulin resistance with expanded islet β-cell mass and hyperinsulinemia [37]. Crossing *Ir^+/−^* mice with mice haploinsufficient for FoxO1 (*Foxo1^+/−^*) improved insulin sensitivity and reduced islet mass [17]. In the current study, we hypothesized that in *Ir^+/−^* mice, increased Gsk-3β as well as FoxO1 activity could be contributing to the insulin-resistant phenotype. We crossed *Ir^+/−^* mice with mice lacking one allele of Gsk-3β (*Gsk-3β^+/−^*) and found that *Gsk-3β^+/−^Ir^+/−^* mice, like *Foxo1^+/−^Ir^+/−^* mice, also had improved insulin sensitivity and reduced β-cell mass. Next, we investigated a mouse that is missing two alleles of the insulin receptor substrate 2 (*Irs2^−/−^*), that is also insulin resistant, but develops profound β-cell destruction resulting in marked diabetes [38]. Although crossing *Irs2^−/−^* mice with *Foxo1^+/−^* mice increased β-cell mass and proliferation [39], suggesting that increased β-cell FoxO1 activity was contributing to β-cell loss in *Irs2^−/−^* mice, we found that Gsk-3β activity in islets of *Irs2^−/−^* mice was also markedly elevated. We determined that *Gsk-3β^+/−^Irs2^−/−^* mice had reduced, but persistent, insulin resistance, yet do not develop diabetes, as a result of maintaining islet β-cell mass. Preservation of β-cell mass in *Gsk-3β^+/−^Irs2^−/−^* mice appeared to be due to accelerated proliferation and decreased apoptosis of β-cells. Reduction of Gsk-3β, like reduction of FoxO1, results in preservation of β-cell mass and rescues the diabetes in this model. The results of these studies now define a new, negatively regulated substrate of the insulin signaling pathway specifically within β-cells that when elevated, can impair replication and increase apoptosis, resulting in loss of β-cells and diabetes.

## Results

### Gsk-3β Deficiency (*Gsk-3β^+/−^*) Promotes Insulin Sensitivity and Reduces the Hyperinsulinemia of Insulin Receptor–Deficient Mice (*Ir^+/−^*)

**Figure 1:**
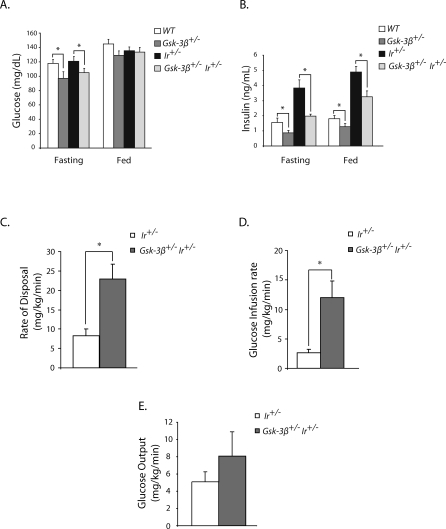
Glucose and Insulin Values in Wild Type (WT), *Gsk-3β^+/−^*, *Ir^+/−^*, or *Gsk-3β^+/−^Ir^+/−^* Mice and Hyperinsulinemic-Euglycemic Clamp in Male *Ir^+/−^* and *Gsk-3β^+/−^Ir^+/−^* Mice Fasting and fed glucose and insulin values in male mice either WT (*n* ≥ 6), haploinsufficient for Gsk-3β (*Gsk-3β^+/−^*; *n* ≥ 6) or the insulin receptor (*Ir^+/−^*; *n* ≥ 6), or combined (*Gsk-3β^+/−^Ir^+/^*; *n* ≥ 6), as indicated. (A) Fasting and fed glucose (26 wk of age). (B) Fasting and fed insulin (26 wk of age). (C) Glucose disposal rate (mg/kg/min). (D) Glucose infusion rate (mg/kg/min). (E) Hepatic glucose output (mg/kg/min) determined on fasted, conscious 28-wk-old mice of indicated genotypes using the hyperinsulinemic-euglycemic clamp (*n* = 4 per each genotype). Results are presented as the mean values ± S.E.M. in the graph. A single asterisk (*) indicates *p* < 0.05.

To determine whether Gsk-3β is a downstream contributor to the insulin resistance of insulin receptor–deficient mice, *Gsk-3β^+/−^* mice were crossed with mice missing one allele of the insulin receptor (*Ir^+/−^*), previously shown to have insulin resistance and elevation of insulin levels in adult animals [37]. Fasting and fed glucose and insulin levels were assessed in *Gsk-3β^+/−^*, *Ir^+/−^*, and compound heterozygous (*Gsk-3β^+/−^Ir^+/−^*) mice and compared to levels in wild type (WT) at 26 wk of age (Figure 1A). Both fasting and fed insulin levels were significantly reduced in mice lacking one allele of Gsk-3β, thus indicating that genetic deficiency of Gsk-3β activity improves insulin sensitivity. In *Ir^+/−^* mice, both fasting and fed insulin levels were higher than in WT mice, consistent with previous reports [37]. In compound heterozygous *Gsk-3β^+/−^Ir^+/−^* mice, the serum insulin values were significantly decreased relative to those in *Ir^+/−^* mice in both the fasting and the fed state, although the values were significantly elevated relative to that in *Gsk-3β^+/−^* mice (*p* < 0.05), suggesting that *Gsk-3β^+/−^Ir^+/−^* mice are still insulin resistant. Similar differences in glucose and insulin values were observed in mice at 8–10 wk of age (Figure S1A and S1B). *Gsk-3β^+/−^Ir^+/−^* mice were found to exhibit improved glucose tolerance relative to that in *Ir^+/−^* mice (Figure S1C). The results of these experiments thus indicate that (1) endogenous Gsk-3β activity contributes to ambient insulin sensitivity, and (2) that Gsk-3β activity is a downstream mediator of the insulin resistance of the *Ir^+/−^* mice.

### *Gsk-3β^+/−^Ir^+/−^* Mice Exhibit Enhanced Peripheral Insulin-Mediated Glucose Disposal and Reduced β-Cell Mass Relative to *Ir^+/−^* Mice

To further characterize the apparent improvement of insulin sensitivity, hyperinsulinemic-euglycemic clamps were performed. The rates of glucose disposal and glucose infusion were increased in *Gsk-3β^+/−^Ir^+/−^* mice relative to those in *Ir^+/−^* mice, confirming enhanced insulin sensitivity (Figure 1C and 1D). Hepatic glucose production did not appear to differ between *Ir^+/−^* mice and *Gsk-3β^+/−^Ir^+/−^* mice (Figure 1E), suggesting that the beneficial effects of genetic reduction of Gsk-3β on carbohydrate metabolism were a result of enhanced effects on peripheral insulin-mediated glucose disposal. These results are consistent with those of Patel et al. (S. Patel, B. W. Doble, K. MacAulay, E. M. Sinclair, D. J. Drucker, and J. R. Woodgett, unpublished data) in which tissue-specific knockout of Gsk-3β in skeletal muscle improved insulin sensitivity, whereas elimination of the gene in liver had no apparent effect on carbohydrate metabolism.

**Figure 2:**
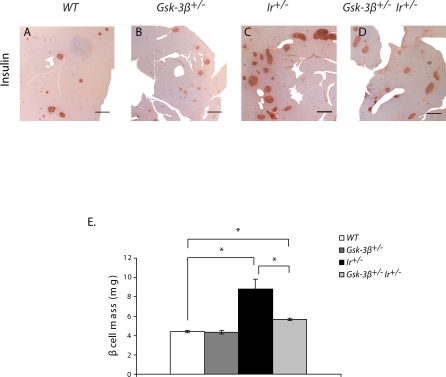
β-Cell Mass in WT, *Gsk-3β^+/−^*, *Ir^+/−^*, or *Gsk-3β^+/−^Ir^+/−^* (A–D) Pancreatic sections from 10-mo-old mice of the indicated genotypes were stained with antibodies to insulin and then were counterstained with hematoxylin. Scale bar represents 100 μm. (E) Random sections of the entire pancreas from 10-mo-old mice of the indicated genotypes were stained with hematoxylin and were measured to determine the β-cell mass (WT: *n* = 3, *Gsk-3β^+/−^*: *n* = 3, *Ir^+/−^*: *n* = 4, and *Gsk-3β^+/−^Ir^+/−^*: *n* = 4). Results are expressed as β-cell mass (mg). All values are represented as the mean ± S.E.M. A single asterisk (*) indicates *p* < 0.05.

Pancreatic sections with insulin staining of each of the four genotypes are shown in Figures 2A–2D. Akthough there were no differences in pancreatic areas (unpublished data), the β-cell mass was increased in *Ir^+/−^* mice as previously noted [37], and reduced in *Gsk-3β^+/−^Ir^+/−^* mice as assessed by pancreatic morphometry (Figure 2E). In conclusion, mice missing one allele of Gsk-3β when crossed with *Ir^+/−^* mice had reduced hyperinsulinemia associated with reduced β-cell mass. 

### Gsk-3β Is Activated in Islets of the Irs2-Deficient (*Irs2^−/−^*) Mice

**Figure 3:**
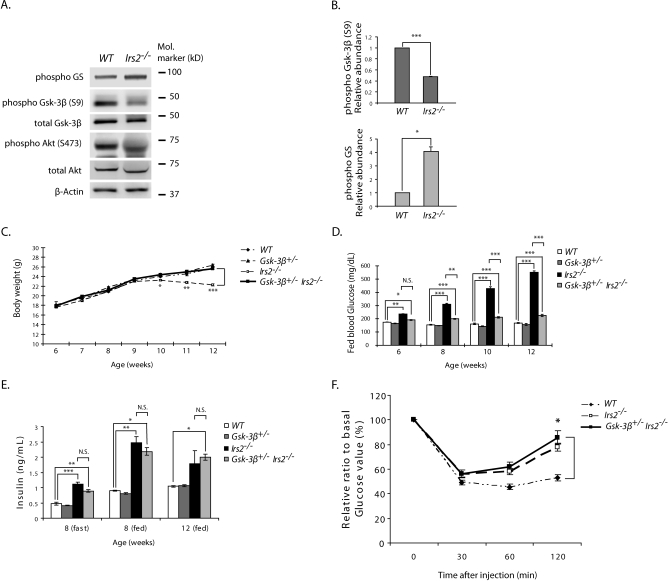
Metabolic Phenotypes of *Irs2^−/−^* and *Gsk-3β^+/−^Irs2^−/−^* Mice (A) Islets were isolated from 6-wk-old WT and *Irs2^−/−^* mice, and were lysed. Lysates were subjected to western blot analysis using anti-phospho Akt (S473), anti-phospho Gsk-3β (S9), anti-total Gsk-3β, anti-phosho glycogen synthase, anti-total Akt, and β-actin. One of four independent experiments is shown. (B) Densitometry of phospho-Gsk-3β (S9) and phospho-glycogen synthase was measured and normalized over total Gsk-3β and β-actin, respectively. Mean phosphorylation ± S.E.M. is represented in the graph. (C) Body weights at indicated age (WT: *n* = 12, *Gsk-3β^+/−^*: *n* = 12, *Irs2^−/−^*: *n* = 29, and *Gsk-3β^+/−^Irs2^−/−^*: *n* = 14). (D) Fed glucose concentration in blood derived from mouse tail vein at indicated age. (WT: *n* = 12, *Gsk-3β^+/−^*: *n* = 12, *Irs2^−/−^*: *n* = 29, and *Gsk-3β^+/−^Irs2^−/−^*: *n* = 14). (E) Fasting and fed plasma insulin levels at 8 wk of age and fed insulin levels at 12 wk of age in mice either WT (*n* = 10), *Gsk-3β^+/−^* (*n* = 9), *Irs2^−/−^* (*n* = 15), or *Gsk-3β^+/−^Irs2^−/−^* (*n* = 12) were determined. (F) Insulin tolerance tests were performed on 4-h fasted male WT (*n* = 4), *Irs2^−/−^* (*n* = 6), and *Gsk-3β^+/−^Irs2^−/−^* (*n* = 4) mice at 6 wk of age. Glucose levels were assessed at the indicated time intervals after intraperitoneal injection of human regular insulin (0.5 U/kg). Results are presented as the mean ± S.E.M. in the graph. A single asterisk (*) indicates *p* < 0.05; double asterisks (**) indicate *p* < 0.01; and triple asterisks (***) indicate *p* < 0.001.

Whereas the *Ir^+/−^* mice have peripheral insulin resistance, Irs2-deficient mice (*Irs2^−/−^*) have both peripheral insulin resistance as well as impaired insulin signaling, as measured by reduced Akt activity in islets [40]. Because Akt is a negative regulator of Gsk-3β activity and Gsk-3β is a known regulator of both proliferation and apoptosis [16], we hypothesized that increased Gsk-3β activity could also contribute to the reduced β-cell mass of *Irs2^−/−^* mice. Islets from *Irs2^−/−^* mice were examined at 6 wk of age and shown to have decreased phosphorylation at serine 473 of Akt and phosphorylation at serine 9 of Gsk-3β in *Irs2^−/−^* compared with WT mice (Figure 3A). Additionally, islets from *Irs2^−/−^* mice were found to have a 4-fold elevation of phosphorylated glycogen synthase, a substrate of Gsk-3β and a measure of its increased activity (Figure 3B).

### Gsk-3β Haplodeficiency in *Irs2^−/−^* Mice Corrects Diabetes

Because of the antiproliferative and proapoptotic effects of Gsk-3β activity in other tissues [16], finding increased Gsk-3β activity in islets from *Irs2^−/−^* mice was consistent with the possibility that this may contribute to the decreased β-cell mass and function of these mice. We therefore crossed mice haploinsufficient for Gsk-3β with *Irs2^−/−^* mice to determine whether it would have beneficial effects on preserving β-cell mass and prevent diabetes. We generated double knockout mice (*Gsk-3β^+/−^Irs2^−/−^*) by interbreeding *Gsk-3β^+/−^* and *Irs2^+/−^* mice. The *Irs2^−/−^* mice gained weight until about 9 wk, when body weight plateaued and began to decline. In contrast, the *Gsk-3β^+/−^Irs2^−/−^* mice continued to increase body weight, indistinguishable from that of the WT or *Gsk-3β^+/−^* mice at 12 wk (Figure 3C) and at 24 wk (Figure S2A).

Fed glucoses were determined at 6, 8, 10, and 12 wk of age (Figure 3D). *Irs2^−/−^* mice developed a progressive increase with severe hyperglycemia, mean glucose >500 mg/dl, at 12 wk of age, confirming previous observations [38]. In contrast, blood glucose concentrations in *Gsk-3β^+/−^Irs2^−/−^* mice were significantly reduced relative to that in *Irs2^−/−^* mice, although plasma insulin did not differ (Figure 3E). Fasting glucose levels at 6 wk of age did not differ, but did at 8 wk (Table S1). To interpret the basis for plasma insulin levels, insulin sensitivity was examined in *Irs2^−/−^* and *Gsk-3β^+/−^Irs2^−/−^* mice relative to that in WT mice by insulin tolerance testing in 6-wk-old mice (Figure 3E). Interestingly, the *Gsk-3β^+/−^Irs2^−/−^* mice maintained insulin resistance relative to that in WT mice, suggesting that the beneficial effects of genetic deficiency of Gsk-3β on restoration of glucose homeostasis is not solely due to altered insulin sensitivity.

### β-Cell Mass Is Preserved in *Gsk-3β^+/−^Irs2^−/−^* Mice

**Figure 4:**
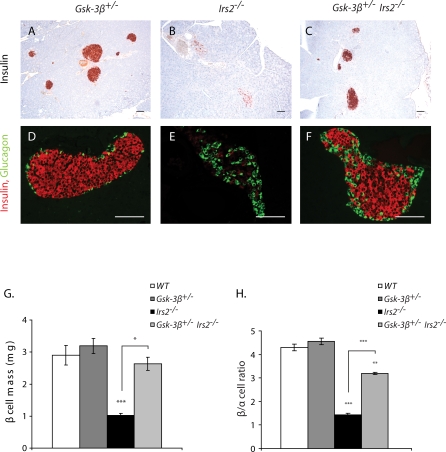
Morphologic Phenotypes of *Irs2^−/−^* and *Gsk-3β^+/−^Irs2^−/−^* Mice (A–C) Pancreatic sections from 8-wk-old mice of the indicated genotypes were stained with antibodies to insulin and then were counterstained with hematoxylin. Scale bar represents 100 μm. (D–F) Pancreatic sections from 8-wk-old mice of the indicated genotypes at 8 wk were stained with antibodies to insulin (red) and glucagon (green). Scale bar represents 50 μm. (G) Random sections of the entire pancreas from 8-wk-old mice of the indicated genotypes were stained with hematoxylin and were measured to determine the β-cell area and quantification of the β cell mass. Results are expressed as the mg of total weight containing insulin-immunoreactive cells. (WT: *n* = 3, *Gsk-3β^+/−^*: *n* = 4, *Irs2^−/−^*: *n* = 6, and *Gsk-3β^+/−^Irs2^−/−^*: *n* = 5). (H) Quantification of the β/α cell ratio is shown. (WT: *n* = 3, *Gsk-3β^+/−^*: *n* = 4, *Irs2^−/−^*: *n* = 5, and *Gsk-3β^+/−^Irs2^−/−^*: *n* = 5). Results are expressed as the mean ± S.E.M.. A single asterisk (*) indicates *p* < 0.05; double asterisks (**) indicate *p* < 0.01; and triple asterisks (***) indicate *p* < 0.001.

Islet morphology in mice of each genotype was assessed at 8 wk of age. Immunostaining for insulin and glucagon on pancreatic sections are shown in Figure 4A–4F. The insulin immunoreactive area in *Irs2^−/−^* mice was severely reduced (Figure 4B and 4E) relative to that in *Gsk-3β^+/−^* mice, consistent with previous reports [38,39]. In contrast, there appeared to be preservation of β-cell mass in the *Gsk-3β^+/−^Irs2^−/−^* mice (Figure 4C and 4F). As quantified in Figure 4G, islet mass of *Irs2^−/−^* mice was reduced to 30% of WT and *Gsk-3β^+/−^* mice, whereas the mass of *Gsk-3β^+/−^Irs2^−/−^* mice did not differ from that of the control mice. Although the α-cell mass was not determined, the reduction in β- to α-cell ratio (Figure 4H) was consistent with the reduction in β-cell mass.

### β-Cell Proliferation Is Maintained and Apoptosis Reduced in Islets of *Gsk-3β^+/−^Irs2^−/−^* Mice

**Figure 5:**
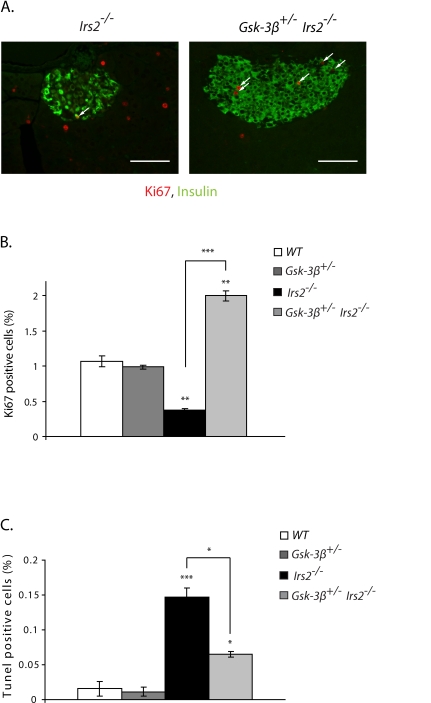
Proliferation and Apoptosis in Islets of the *Irs2^−/−^* and *Gsk-3β^+/−^Irs2^−/−^*  Mice (A) Pancreatic sections from 8-wk-old mice of the indicated genotypes were stained with antibodies to Ki67 (red) and insulin (green). Arrowheads indicate proliferating cells. Scale bar represents 50 μm. (B) The number of cells that are positive for both Ki67 and insulin have been quantified as a percentage of total number of insulin-positive cells in the sections. (WT: *n* = 3, *Gsk-3β^+/−^*: *n* = 3, *Irs2^−/−^*: *n* = 5, and *Gsk-3β^+/−^Irs2^−/−^*: *n* = 5). Results are represented as mean ± S.E.M. Double asterisks (**) indicate *p* < 0.01. (C) Apoptotic β-cells in pancreatic sections from 8-wk-old mice of the indicated genotypes (WT: *n* = 4, *Gsk-3β^+/−^*: *n* = 4, *Irs2^−/−^*: *n* = 7, and *Gsk-3β^+/−^Irs2^−/−^*: *n* = 5) were assessed through TUNEL and anti-insulin staining. The proportion of cells that are positive for both are represented as a percentage of the total number of insulin-positive cells in the sections. Results are represented as mean ± S.E.M. A single asterisk (*) indicates *p* < 0.05; triple asterisks (***) indicate *p* < 0.001.

The progressive loss of β-cell mass in *Irs2^−/−^* mice has been shown to be associated with reduced proliferation and increased apoptosis [38–41]. Preservation of β-cell mass in the *Gsk-3β^+/−^Irs2^−/−^* mice relative to that in the *Irs2^−/−^* mice could be due to increased proliferation or reduced apoptosis or both. We measured Ki67-positive cells in β-cells to assess proliferation in *Irs2^−/−^* and *Gsk-3β^+/−^Irs2^−/−^* mice, as shown in Figure 5A. The percent of Ki67-positive β-cells in *Irs2^−/−^* mice was markedly reduced compared to that in WT and *Gsk-3β^+/−^* mice at 8 wk of age (Figure 5B). Remarkably, the percentage of Ki67-positive cells in *Gsk-3β^+/−^Irs2^−/−^* mice was over four times greater than in *Irs2^−/−^* mice, and even increased approximately 2-fold relative to that in WT and *Gsk-3β^+/−^*. We next assessed apoptosis by terminal deoxynucleotidyl transferase-mediated dUTP-biotin nick-end labeling (TUNEL) staining in pancreatic β-cells at 8 wk of age (Figure S3). The number of TUNEL-positive β-cells was markedly increased in *Irs2^−/−^* mice compared to that in WT and *Gsk-3β^+/−^* mice (Figure 5C). Whereas TUNEL positivity was significantly increased in *Gsk-3β^+/−^Irs2^−/−^* mice relative to that in WT and *Gsk-3β^+/−^* mice, importantly there was approximately a 60% reduction in TUNEL-positive cells in *Gsk-3β^+/−^Irs2^−/−^* mice compared to *Irs2^−/−^* mice. These observations indicate that the preservation of β-cell mass in *Gsk-3β^+/−^Irs2^−/−^* mice was associated with reversal of decreased proliferation and increased apoptosis of *Irs2^−/−^* mice.

### Pdx1 Levels Are Maintained and p27^kip1^ Levels Are Reduced in Islets from *Gsk-3β^+/−^Irs2^−/−^* Mice

**Figure 6:**
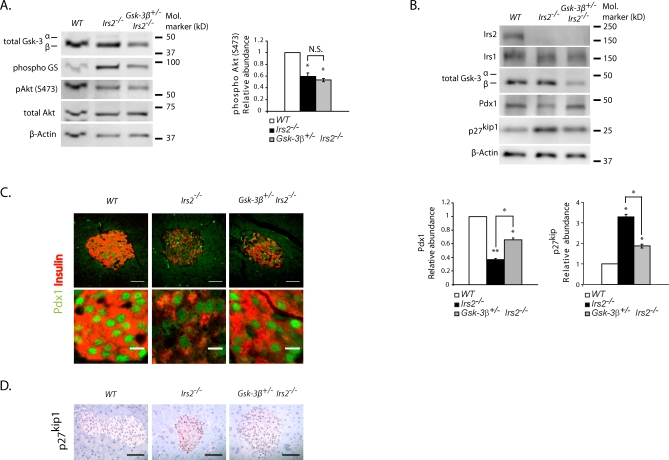
Effects of Inhibition of Gsk-3β Activity on Pdx1 and p27^kip1^ Expression in Islets from *Irs2^−/−^* Mice Islets were isolated from 6 wk-old WT, *Gsk-3β^+/−^Irs2^−/−^*, and *Irs2^−/−^* mice. (A) Western blot analysis with anti-total Gsk-3, anti-phospho glycogen synthase, anti-phospho Akt (S473), anti-total Akt, and β-actin. Representative results of four independent experiments are presented. Densitometry of phosphorylation of Akt (S473) was measured and normalized over total Akt. Mean values ± S.E.M. are summarized on the graph. A single asterisk (*) indicates *p* < 0.05. (B) Western blot analysis with anti-total Gsk-3, anti-Irs2, anti-Irs1, anti-Pdx1, anti-p27^kip1^, and anti-β-actin. Representative results of four independent experiments are presented. Densitometry of Pdx1 and p27^kip1^ was measured and normalized over β-actin. Mean protein levels ± S.E.M. are summarized on the graph. A single asterisk (*) indicates *p* < 0.05; double asterisks (**) indicate *p* < 0.01. (C) Pancreatic sections from 8-wk-old mice of the indicated genotypes at 8 wk were stained with antibodies to Pdx1 (green) and insulin (red). Scale bars represent 50 μm (top) and 10 μm (bottom). (D) Immunostaining for p27^kip1^ in pancreatic islets of 8-wk-old WT, *Irs2^−/−^*, and *Gsk-3β^+/−^Irs2^−/−^* mice. Hematoxylin counterstaining reveals nucleus in dark blue (and acinar cells in light blue); p27^kip1^ staining is in red. Scale bars represent 50 μm.

To examine possible molecular mechanisms for the preservation of islet β-cell mass in *Gsk-3β^+/−^Irs2^−/−^* mice, islets from WT, *Irs2^−/−^*, and *Gsk-3β^+/−^Irs2^−/−^* mice at 6 wk of age were examined by western blot analysis to assess insulin signaling upstream and downstream of Gsk-3β. Phosphorylation of Akt was equally reduced in *Irs2^−/−^* and *Gsk-3β^+/−^Irs2^−/−^* mice (Figure 6A), indicating that haploinsufficiency for Gsk-3β did not alter the insulin signaling pathway through Akt. Interestingly, total Gsk-3 activity remained reduced in *Gsk-3β^+/−^Irs2^−/−^*, indicating that neither the intact Gsk-3β allele nor the Gsk-3α alleles compensated to increase Gsk-3 activity. *Gsk-3β^+/−^Irs2^−/−^* mice had no change in Irs1 expression levels relative to those in *Irs2^−/−^* mice (Figure 6B). Loss of β-cells in *Irs2^−/−^* mice was shown to be associated with decreased islet Pdx1 protein [39,42], whereas transgenic expression of Pdx1 rescued the diabetic phenotype [43]. Associated with preservation of β-cell mass, *Gsk-3β^+/−^Irs2^−/−^* mice had preservation of Pdx1 levels relative to that in *Irs2^−/−^* mice by western blot analysis (Figure 6B) and nuclear localization by immunocytochemistry (Figure 6C).

The *Irs2^−/−^* mice have been shown to have increased levels of the cyclin-dependent kinase inhibitor p27^kip1^ in β-cells, and the loss of β-cell mass and diabetes was corrected when these mice were placed on a p27^kip1^ null background [40]. This established p27^kip1^ as a rate-limiting determinant of proliferation in this insulin-resistant model. Interestingly, it was recently shown that in non–β-cells, Gsk-3β stabilizes p27^kip1^ [44]. We confirmed that islets of *Irs2^−/−^* mice have increased p27^kip1^ protein, and found that the increased proliferation in β-cells of *Gsk-3β^+/−^Irs2^−/−^* mice was associated with decreased p27^kip1^ levels (Figure 6B). Immunostaining on islets of WT mice revealed that only some of the islet cells were positive for p27^kip1^ (Figure 6D, left). In contrast, virtually all of the nuclei of the islet cells in *Irs2^−/−^* mice were positive (Figure 6D, middle). In *Gsk-3β^+/−^Irs2^−/−^* mice, p27^kip1^ could only be detected in some of the nuclei of the islet cells (Figure 6D, right), with a staining that appears to be weaker than observed in *Irs2^−/−^* mice, consistent with the increased proliferation observed in these mice.

### β-Cell–Specific Gsk-3β Deficiency in *Irs2^−/−^* Mice Corrects Diabetes

**Figure 7:**
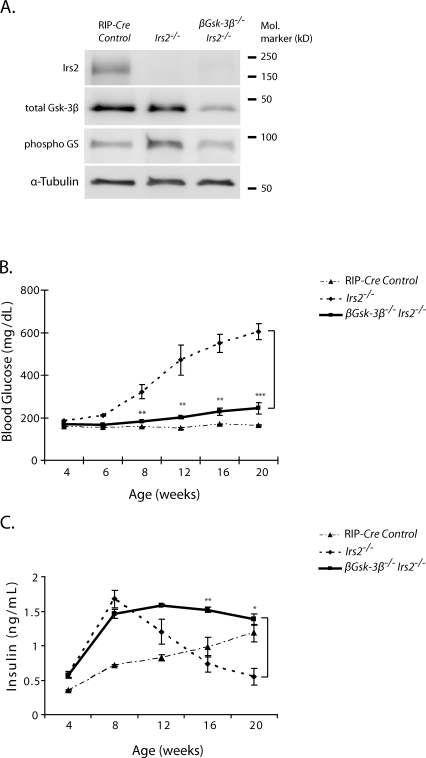
The Effects of β-Cell–Specific Gsk-3β Deficiency in *Irs2^−/−^* Mice (A) Islets were isolated from 6-wk-old control mice, *Irs2^−/−^* mice, and *βGsk-3β^−/−^Irs2^−/−^* mice were lysed. Lysates were subjected to western blot analysis using anti-Irs2, anti-total Gsk-3, anti-phosho glycogen synthase, and α-tubulin. One of two independent experiments is shown. (B) Fed glucose levels were assessed in control mice (filled triangles; *n* = 11), *Irs2^−/−^* mice (filled diamonds; *n* = 10), and *βGsk-3β^−/−^ Irs2^−/−^* mice (filled squares; *n* = 5) at the indicated ages. (C) Fed plasma insulin levels were assessed in control mice (filled triangles; *n* = 11), *Irs2^−/−^* mice (filled diamonds; *n* = 10), and *βGsk-3β^−/−^ Irs2^−/−^* mice (filled squares; *n* = 5) at the indicated ages. Results are presented as the mean ± S.E.M. in the graph. A single asterisk (*) indicates *p* < 0.05; double asterisks (**) indicate *p* < 0.01; and triple asterisks (***) indicate *p* < 0.001.

To separate peripheral versus β-cell–specific effects of reduction of Gsk-3β activity on preservation of β-cell mass, mice homozygous for a floxed *Gsk-3β* allele (*Gsk-3β^F/F^*) were then crossed with rat insulin 2 promoter-Cre (*RIP-Cre*) mice to produce β-cell–specific knockout of Gsk-3β (*βGsk-3β^−/−^*). As shown in Figure 7A, expression of islet Gsk-3β was reduced more than 80%. *βGsk-3β^−/−^Irs2^−/−^* mice also maintained relatively normal plasma glucose compared to the severe hyperglycemia of the *Irs2^−/−^* mice for the 20 wk of observation (Figures 7B). Additionally, the hyperinsulinemia of the *Irs2^−/−^* mice was maintained in *βGsk-3β^−/−^Irs2^−/−^* mice (Figure 7C).

### Gsk-3 Activity Stabilizes p27^kip1^ in Mouse Insulinoma Cells and in Primary Mouse Islets

**Figure 8:**
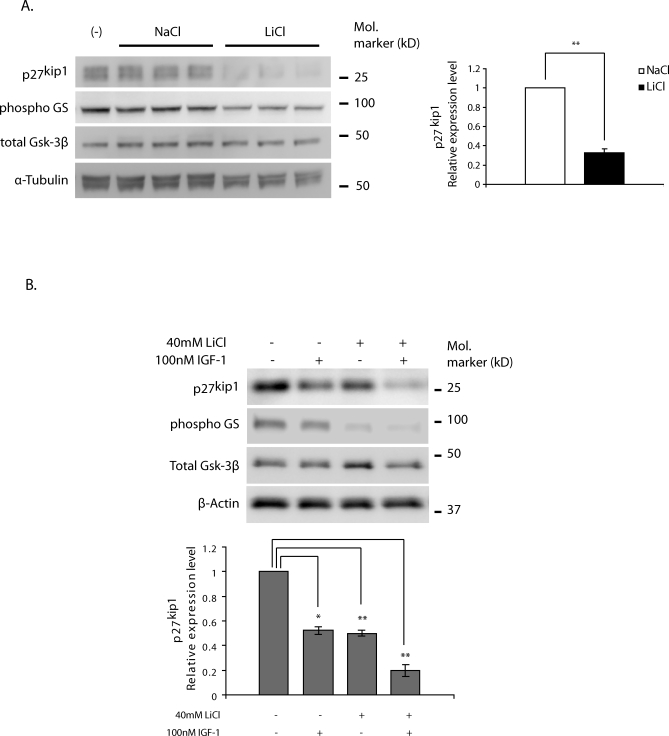
Effects of Inhibition of Gsk-3 Activity on Protein Stability of p27^kip1^ (A) MIN6 cells were pretreated with either 40 mM lithium chloride (LiCl) or 40 mM NaCl in DMEM with 15% FBS for 1 h and were cotreated with 25 μg/ml cyclohexamide and lithium or NaCl for 4 h. The lysates were subjected to western blot analysis with anti-p27^kip1^, phospho-glycogen synthase, total Gsk-3β, and α-tubulin. Densitometry of p27^kip1^ was measured and normalized over α-tubulin. (B) Islets isolated from 16-wk-old WT mice were deprived of serum for 4 h and were incubated with no addition, or with addition of 100 nM IGF-1, or 40 mM LiCl, or both for 3 h. Lysates were then prepared from islets and were subjected to western blot analysis with anti-p27^kip1^, anti-phospho glycogen synthase, anti-total Gsk-3β, and β-actin. Representative results of three independent experiments are presented. Densitometry of p27^kip1^ was measured and normalized over β-actin. Mean protein levels ± S.E.M. are summarized on the graph. A single asterisk (*) indicates *p* < 0.05; double asterisks (**) indicate *p* < 0.01.

To further examine effects of reduction in Gsk-3 activity on protein stability of p27^kip1^, mouse insulinoma cells were pretreated with lithium to inhibit Gsk-3 activity, and protein levels were assayed 4 h after addition of cyclohexamide to inhibit new protein synthesis. Lithium treatment reduced Gsk-3 activity and markedly reduced levels of p27^kip1^ compared to cells treated with NaCl as an osmotic control (Figure 8A).

To confirm the physiological significance of the effects of Gsk-3 activity on p27^kip1^ levels, primary mouse islets were examined. Islets were isolated from WT mice and incubated for 4 h in serum-free medium, followed by 3 h with no addition, or with the addition of either IGF-1 to activate the insulin signaling pathway, with lithium, or with both, or islet protein lysates blotted for p27^kip1^ levels (Figure 8B). Addition of IGF-1 and lithium resulted in reduced p27^kip1^ levels, with maximum reduction with addition of both. These results provide physiological support for the conclusion that Gsk-3 activity stabilizes p27^kip1^ levels in pancreatic islets.

## Discussion

The current study offers specific genetic approaches to assess the role of Gsk-3β in control of β-cell mass in insulin-resistant diabetic models, and as a consequence, several novel observations were made. Loss of one allele of Gsk-3β in WT mice promotes insulin sensitivity and in *Ir^+/−^* mice reduces insulin resistance and improves glucose tolerance by enhancing glucose disposal. Severely insulin-resistant *Irs2^−/−^* mice were found to have elevated islet Gsk-3 activity associated with severe reduction of β-cell proliferation and elevated apoptosis. Loss of one allele of Gsk-3β in *Irs2^−/−^* mice reversed these findings, preserving β-cell mass and preventing diabetes. Additionally, Pdx1 levels were depressed and p27^Kip1^ levels were increased in islets of *Irs2^−/−^* mice, and they were also reversed by loss of one allele of Gsk-3β. β-cell–specific deficiency of Gsk-3β reversed the diabetes of the *Irs2^−/−^* mice, indicating the importance of Gsk-3β in islet β-cells. Finally, in vitro studies demonstrated that Gsk-3 activity stabilizes p27^kip1^ levels, suggesting a mechanism for impairment of proliferation. The results of these studies thus indicate that in insulin-resistant animals, Gsk-3β impairs replication and enhances cell death, leading to postnatal β-cell loss and diabetes.

FoxO1 and Gsk-3β are both negatively regulated targeted proteins of the insulin/PI-3K/Akt signaling pathway. Previous studies showed that *Irs2^−/−^* mice crossed with *Foxo1^+/−^* mice resulted in partial correction of fed plasma glucose, β-cell mass, and proliferation [39], along with improved Pdx1 expression. In the current study, we found that islets of *Irs2^−/−^* mice had increased Gsk-3 activity (Figure 3A and 3B), now demonstrating that both Gsk-3 and FoxO1 significantly contribute to the impaired proliferation and increased apoptosis in *Irs2^−/−^* mice.

What are the mechanisms that could account for postnatal loss of β-cell mass in the insulin-resistant models? Evidence to date suggests that FoxO1 is contributing through impaired proliferation and enhanced apoptosis via transcriptional mechanisms, as it has been shown to repress Pdx1 transcription in insulinoma cells [39]. In non–β-cells FoxO1 has also been shown to increase p27^Kip1^ expression [45]. The results of the current studies suggest a novel mechanism for regulation of Pdx1 and p27^Kip1^ levels in insulin-resistant β-cells through Gsk-3β activity. In non–β-cells, the half-life (*t*_1/2_) of p27^Kip1^ protein was 12 h in the absence of growth factors, and 20 min when growth factors were restored, or when cells were treated with Gsk-3β inhibitors [44]. Gsk-3β phosphorylated and stabilized p27^Kip1^, whereas Gsk-3β inhibitors targeted p27^Kip1^ for proteosomal degradation. These results suggest a possible mechanism by which Gsk-3 activity might regulate cell proliferation in β-cells through altered p27^Kip1^ stability. There is substantial evidence that this mechanism is operational in β-cells. First, p27^kip1^ levels were increased in islets from *Irs2^−/−^* mice (Figure 6B and 6D, and [40]), and levels were reduced with elimination of one allele of Gsk-3β. Second, in vitro evidence is presented showing that Gsk-3 activity regulates p27^Kip1^ protein stability in insulinoma cells (Figure 8A). Third, physiological support for the conclusion that Gsk-3 activity stabilizes p27^kip1^ levels in pancreatic islets is presented (Figure 8B). In contrast to p27^Kip1^ protein stabilization, Gsk-3 activity was shown to destabilize Pdx1 protein levels in insulinoma cells [46]. We have confirmed this effect of Gsk-3 activity on Pdx1 in MIN6 cells (unpublished data), and now provide in vivo evidence that reduction of islet Gsk-3β activity restores Pdx1 levels in islets of *Gsk-3β^+/−^Irs2^−/−^* mice (Figure 6B and 6C).

The *Irs2^−/−^* mice have severe impairment of the insulin signaling PI-3K/Akt pathway, and rapidly lose β-cell mass. Elimination of one allele of Gsk-3β in *Irs2^−/−^* mice preserves β-cell mass and, for the most part, maintains glucose homeostasis, yet Gsk-3β is only one of many substrates regulated by the insulin signaling pathway. For example, *Irs2^−/−^* mice have increased FoxO1 [39], and perhaps decreased S6K levels, along with alterations in other Akt substrates. Although the *Gsk-3β^+/−^Irs2^−/−^* mice maintain apparently normal β-cell mass, they are still insulin resistant, and therefore not fully functional; they would be anticipated to have expanded β-cell mass as shown in the *Ir^+/−^* mice (Figure 2E). Thus Gsk-3β is only one protein among many necessary for fully functional β-cells.

Elimination of one allele of Gsk-3β in insulin-resistant *Ir^+/−^* mice enhanced insulin sensitivity by augmenting peripheral insulin-mediated glucose disposal, independent of effects on hepatic glucose output (Figure 1C–1E). These results are consistent with those in which tissue-specific knockout of Gsk-3β in skeletal muscle enhanced insulin sensitivity (S. Patel, B. W. Doble, K. MacAulay, E. M. Sinclair, D. J. Drucker, and J. R. Woodgett, unpublished data). Could enhanced peripheral insulin sensitivity by loss of one allele of Gsk-3β account for the preservation of β-cell mass in the *Irs2^−/−^* mice? The results with conditional knockout of the Gsk-3β gene in β-cells indicate the importance of this protein in the β-cell with impaired insulin signaling and that under these circumstances, Gsk-3α is not playing a major role. Although the exact contribution of Gsk-3α to β-cell function has yet to be determined, the results of Patel et al. (S. Patel, B. W. Doble, K. MacAulay, E. M. Sinclair, D. J. Drucker, and J. R. Woodgett, unpublished data) and MacAulay et al. [47] emphasize the isoform and tissue-selective effects of the two mammalian Gsk-3s in skeletal muscle and liver.

The results of these studies now define a new, negatively regulated substrate of the insulin signaling pathway specifically within β-cells that when elevated, can impair replication and increase apoptosis, resulting in postnatal loss of β-cells and diabetes. These results thus form the rationale for developing agents to inhibit this enzyme in obese insulin-resistant individuals to preserve β-cells and prevent diabetes onset.

## Materials and Methods

### Animal production and phenotypic analysis.

Generation and genotyping of *Gsk-3β^+/−^*, *Ir^+/−^*, and *Irs2^−/−^* mice have been described [23,37,38,48]. We maintained *Ir^+/−^* mice on the C57BL/6J background and the *Irs2^−/−^* mice on a mixed C57BL/6J × 129Sv background, and crossed them with *Gsk-3β^+/−^* mice on the C57BL/6J × 129Sv background to obtain *Gsk-3β^ +/−^Ir^+/−^* and *Gsk-3β^+/−^Irs2^+/−^* mice on a mixed C57BL/6J × 129Sv background. Double-heterozygote F_1_ offspring were intercrossed (*Gsk-3β^+/−^Irs2^+/−^*) to obtain *Gsk-3β^+/−^Irs2* ^−/−^ mice. The *Gsk-3β^+/−^Irs2^−/−^* progeny was observed at the expected Mendelian frequency in both instances. WT control mice have been obtained from littermates of double-heterozygous breeding.

The generation of mice expressing a conditional allele of Gsk-3β will be described in further detail (S. Patel, B. W. Doble, K. MacAulay, E. M. Sinclair, D. J. Drucker, and J. R. Woodgett, unpublished data). In brief, R1 embryonic stem (ES) cells were electroporated with a modified Gsk-3β targeting vector whereby LoxP sites were introduced by PCR into the intronic region flanking exon 2 of Gsk-3β, and a neomycin resistance cassette was inserted and flanked by FLP recombinase target (FRT) sites

ES cell clones that had undergone correct homologous recombination were identified by Southern blot and microinjected into C57/B6J blastocysts. The resultant chimeric mice were crossed to C57/B6J, and germline transmission of the Gsk-3β floxed allele was verified by PCR. Resultant interbreeding of these mice yielded Gsk-3β floxed mice that were viable, healthy, born with the expected Mendelian frequency, and expressed Gsk-3β at levels indistinguishable from WT animals.

Pancreatic β-cell–specific Gsk-3β knockout mice (*βGSK-3β^–/–^*) were generated by breeding Gsk-3β floxed mice with mice that express the Cre recombinase gene under the control of the promoter of the rat insulin 2 gene [49]. To obtain *βGsk-3β^–/–^Irs2^+/−^* mice, F_1_ offspring (*Gsk-3β^F/+^Irs2^+/−^*) were intercrossed. They were then further intercrossed with F_2_ offspring (*βGsk-3β^–/–^Irs2^+/−^*) to obtain *βGsk-3β^–/–^Irs2^−/−^* mice.

Blood glucose as well as serum insulin concentrations were determined as previously described [13]. For the glucose tolerance test, mice were subjected to an overnight fast followed by intraperitoneal glucose injection (2.0 g/kg). Blood samples were collected at 0, 30, 60, and 120 min after the injection. For the insulin tolerance tests, mice were subjected to a 4-h fast followed by intraperitoneal human regular insulin injection (0.5 U/kg). Blood samples were collected 0, 30, 60, and 120 min after the injections. After sacrificing the mice, the pancreas was removed and weighed. All experiments were carried out with male mice. This project was approved by the Animal Ethics Committee of Washington University School of Medicine.

### Hyperinsulinemic-euglycemic clamps.

Clamp experiments were essentially performed as previously described [50,51]. Double-lumen catheters were placed and 3-[3H] glucose was infused to steady state. Regular human insulin was infused at 20 mU/kg per min with 25% d-glucose to maintain the blood glucose at 120 mg/dl for at least 90 min. The 3-[3H] glucose infusion was continued during the clamp, with labeled glucose included in the 25% d-glucose infusion to match blood specific activity at steady state. The rate of appearance of glucose (Ra), equal to the rate of glucose utilization (Rd) at steady state, was determined by dividing the infusion rate of labeled glucose by the specific activity. Endogenous glucose production was calculated by subtracting the cold glucose infusion rate from the clamp Rd.

### Immunohistochemical and morphometric analysis of the pancreatic islets.

Pancreas were isolated and fixed from 8-wk-old *Irs2^−/−^*, *Gsk-3β^+/−^Irs2^−/−^*, and *Gsk-3β^+/−^* mice. Isolated pancreas were fixed overnight in 3.7% formaldehyde at room temperature. Tissue was then routinely processed for paraffin embedding, and 5-μm sections were cut and mounted on glass slides. The sections were immunostained with antibodies to insulin (Dako), glucagon (Sigma Aldrich), Ki67 (Zymed Laboratories/Invitrogen), Pdx-1 (Joel Habener), and p27^kip1^ (BD Transduction Laboratories). Histomouse-SP (Zymed Laboratories/Invitrogen) was used for secondary antibodies for brightfield microscopy and β-cell mass, whereas Cy3- or fluorescein isothiocyanate−conjugated (FITC) secondary antibodies (Jackson Immunoresearch) were used for fluorescence microscopy. All images were acquired on a DM4000 B microscope (Leica Microsystems). The β-cell area was determined after the analysis of a number of random sections stained for insulin and analyzed with NIH Image 1.38x software [52]. The total β-cell mass was then calculated using the following calculation: [(islet area/total pancreas area) × pancreas weight]. Five pancreatic sections from each animal, including representative sections of pancreas, and at least 100 islets per mouse were counted. Adjacent nonoverlapping fields were analyzed to obtain a true representation of average islet/β cell distribution throughout the pancreas.

TUNEL staining was performed on pancreatic sections using the ApopTag in situ apoptosis detection kit and according to the manufacturer's instructions (Chemicon/Milipore). At least 3,000 insulin-positive cells were counted for each mouse to assess the percentage of TUNEL-positive cells among insulin-positive cells.

Quantitative data are obtained from at least three mice in each group, unless indicated.

### Western blotting analysis.

Islet isolation were carried out as described previously [53]. For immunoblot analysis, isolated islets were lysed in ice-cold cell lysis buffer consisting of 50 mM HEPES (pH 7.5), 1% (v/v) Nonidet P-40, 2 mM activated sodium orthovanadate, 100 mM sodium fluoride, 10 mM sodium pyrophosphate, 4 mM EDTA, 1 mM phenylmethylsulfonyl fluoride, 1 μg/ml leupeptin, and 1 μg/ml aprotinin, then passed through a syringe ten times; particulate material was removed by centrifugation (10,000 × g; 10 min; 4 °C). The supernatant was collected. The extracts (50 μg of total protein) were subjected to immunoblot analysis with antibodies to Gsk-3β phosphorylated on Ser9, Akt phosphorylated on Ser473, Akt phosphorylated on Thr308, total Akt, total Gsk-3α/β (Cell Signaling), total Gsk-3β (BD Transduction Laboratories), and glycogen synthase phosphorylated on Ser 641 and 645 (Biosource/Invitrogen), Irs2 (Upstate), Pdx1 (Santa Cruz SC14664), p27^kip1^ (BD Transduction Laboratories), α-tubulin, and β-actin (Sigma-Aldrich).

For immunoblot analysis with mouse insulinoma MIN6 cells, lysates of MIN6 cells treated with either 40 mM lithium or 40 mM NaCl for 1 h, then cotreated with 25 μg/ml cyclohexamide and lithium for 4 h, were prepared. The lysates were probed with the antibodies listed above.

Islets isolated from 16-wk-old WT mice were deprived of serum for 4 h and were incubated in the absences or presence of 10% FBS, 100 nM IGF-1, or 40 mM LiCl for 3 h. Lysates were then prepared from islets and were subjected to western blot analysis with the antibodies listed above.

Immune complexes were revealed using ECL Advance Western Blot Detection kit (Amersham), and the images were acquired using a FluoroChemi 8800 digital camera acquisition system (Alpha Innotech). Band intensities in the blots were later quantified using ImageJ 1.39j [53] and α-tubulin or β-actin bands were used to adjust for loading differences.

### Statistical analysis.

Quantitative data are presented as the mean ± the standard error of the mean (S.E.M.) from at least three independent experiments and at least 100 islets from more than three mice, unless indicated. We assessed interactions among variables by two-way analysis of variance and used the Student' *t*-test to compare independent means. A *p*-value of 0.05 was considered statistically significant.

## Supporting Information

Figure S1 The Effects of Haploinsufficiency for Gsk-3β on Carbohydrate Metabolism in *Ir^+/−^* Mice at 8–10 wk of Age (A) Fasting and fed glucose of indicated genotypes at (8–10 wk of age).
(B) Fasting and fed insulin levels (8–10 wk of age).
(C) Intraperitoneal glucose tolerance tests were performed on overnight-fasted male *Ir^+/−^* mice at 8 wk of age and *Gsk-3β^+/−^Ir^+/−^* mice at 8 wk of age after intraperitoneal injection of d-glucose (2 g/kg of body weight; *n* = 6). Glucose levels were assessed at the indicated time intervals. Results are presented as the mean ± S.E.M. in the graph. A single asterisk (*) indicates *p* < 0.05.
(266 KB PDF)

Figure S2 The Effects of Haploinsufficiency for Gsk-3β on Carbohydrate Metabolism in *Irs2^−/−^* Mice at 24 wk of Age (A) Body weight in mice, either WT (*n* = 10) or *Gsk-3β^+/−^Irs2^−/−^* (*n* = 13).
(B) Fed glucose in mice, either WT (*n* = 10) or *Gsk-3β^+/−^Irs2^−/−^* (*n* = 13).
(C) Fed insulin levels in mice, either WT (*n* = 7) or *Gsk-3β^+/−^Irs2^−/−^* (*n* = 8). Results are presented as the mean ± S.E.M. in the graph. A single asterisk (*) indicates *p* < 0.05.
(231 KB PDF)

Figure S3 The Effects of Haploinsufficiency for Gsk-3β on Apoptosis in the Islets of *Irs2^−/−^* Mice Pancreatic sections from 8-wk-old mice of indicated genotypes were subjected to TUNEL assay. Arrowheads indicate apoptotic nuclei. Scale bar represents 50 μm.
(7.8 MB PDF)

Table S1 Fasting Blood Glucose Concentration at 6 and 8 wk of Age in Either WT, *Gsk-3β^+/−^*, *Irs2^−/−^*, or *Gsk-3β^+/−^Irs2^−/−^* Mice Fasting glucose in mice, either WT (*n* = 10), *Gsk-3β^+/−^* (*n* = 9), *Irs2^−/−^* (*n* = 15), or *Gsk-3β^+/−^Irs2^−/−^* (*n* = 12) were determined after an overnight fast at 6 and 8 wk of age. Mean values ± S.E.M. are summarized in the table. Triple asterisks (***) indicate *p* < 0.001.
(216 KB PDF)

## Abbreviations

Gsk: glycogen synthase kinase
Ir: insulin receptor
Irs: insulin receptor substrate
S.E.M.: standard error of the mean
TUNEL: terminal deoxynucleotidyl transferase-mediated dUTP-biotin nick-end labeling
WT: wild type
