# Supplementary material for: Genetic Deficiency of Glycogen Synthase Kinase-3β Corrects Diabetes in Mouse Models of Insulin Resistance
Source: PLoS Biol. 2008 Feb 19;6(2):e37. doi: 10.1371/journal.pbio.0060037 (PMC2245985; doi:10.1371/journal.pbio.0060037)
Supplement: Supplementary file 1 [file pbio.0060037.sg001.pdf]

S1

A.

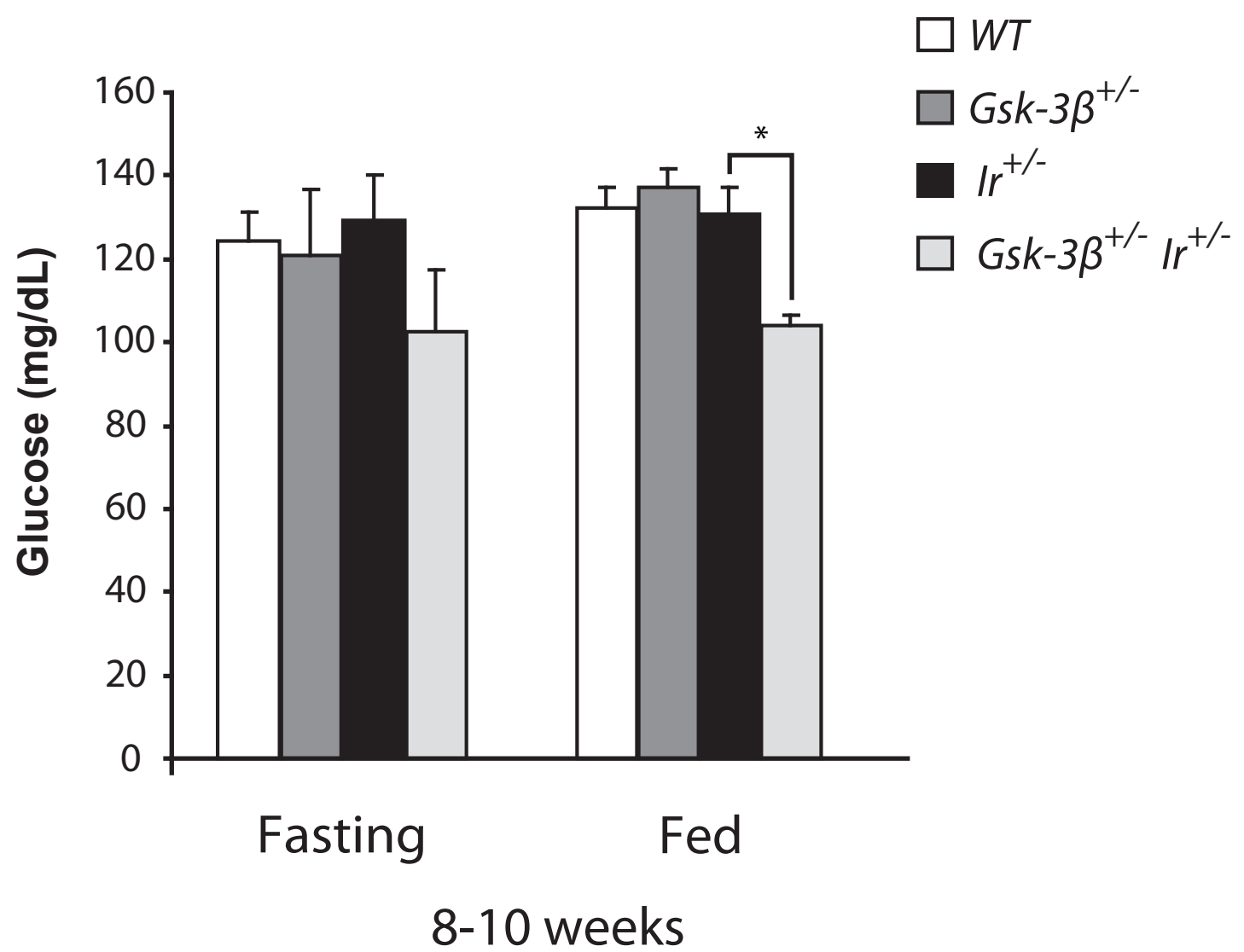
WT
Gsk-3β+/-
Ir+/-
Gsk-3β+/- Ir+/-
Glucose (mg/dL)
160
140
120
100
80
60
40
20
0
*
Fasting
Fed
8-10 weeks

B.

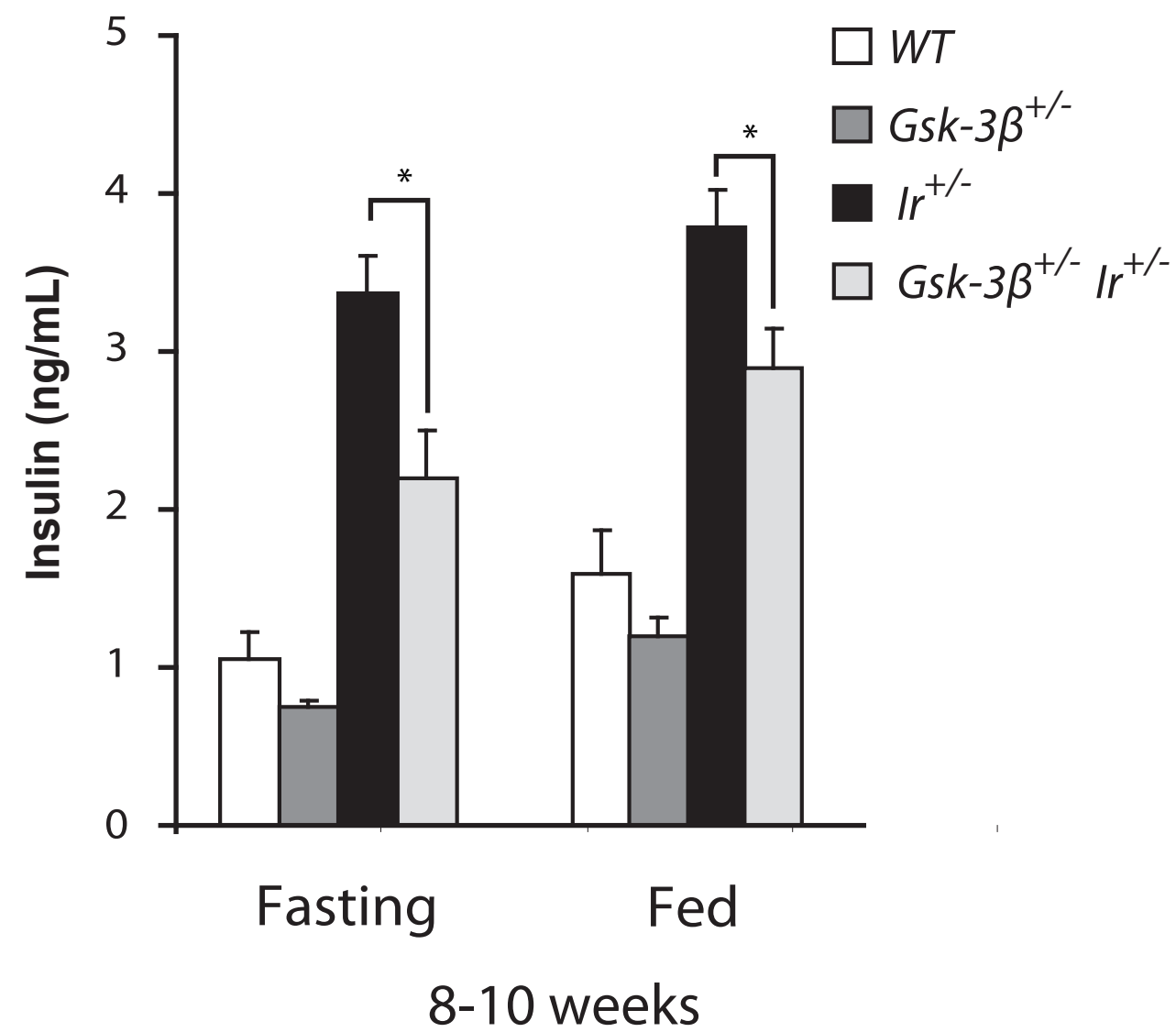
WT
Gsk-3β+/-
Ir+/-
Gsk-3β+/- Ir+/-
Insulin (ng/mL)
5
4
3
2
1
0
*
*
Fasting
Fed
8-10 weeks

C.

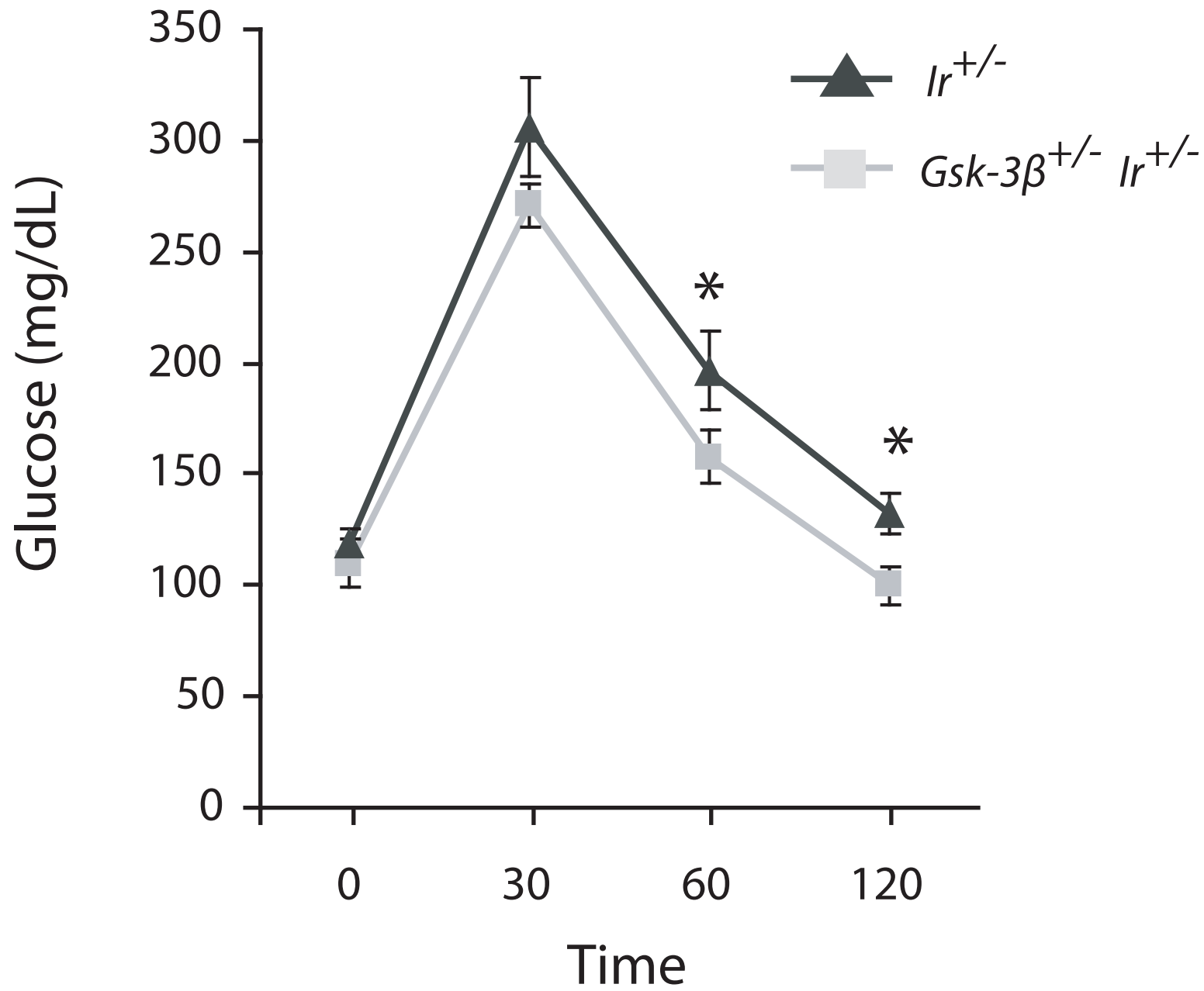
Ir+/-
Gsk-3β+/- Ir+/-
Glucose (mg/dL)
350
300
250
200
150
100
50
0
*
*
0
30
60
120
Time
