# Supplementary material for: Genetic Deficiency of Glycogen Synthase Kinase-3β Corrects Diabetes in Mouse Models of Insulin Resistance
Source: PLoS Biol. 2008 Feb 19;6(2):e37. doi: 10.1371/journal.pbio.0060037 (PMC2245985; doi:10.1371/journal.pbio.0060037)
Supplement: Supplementary file 2 [file pbio.0060037.sg002.pdf]

S2

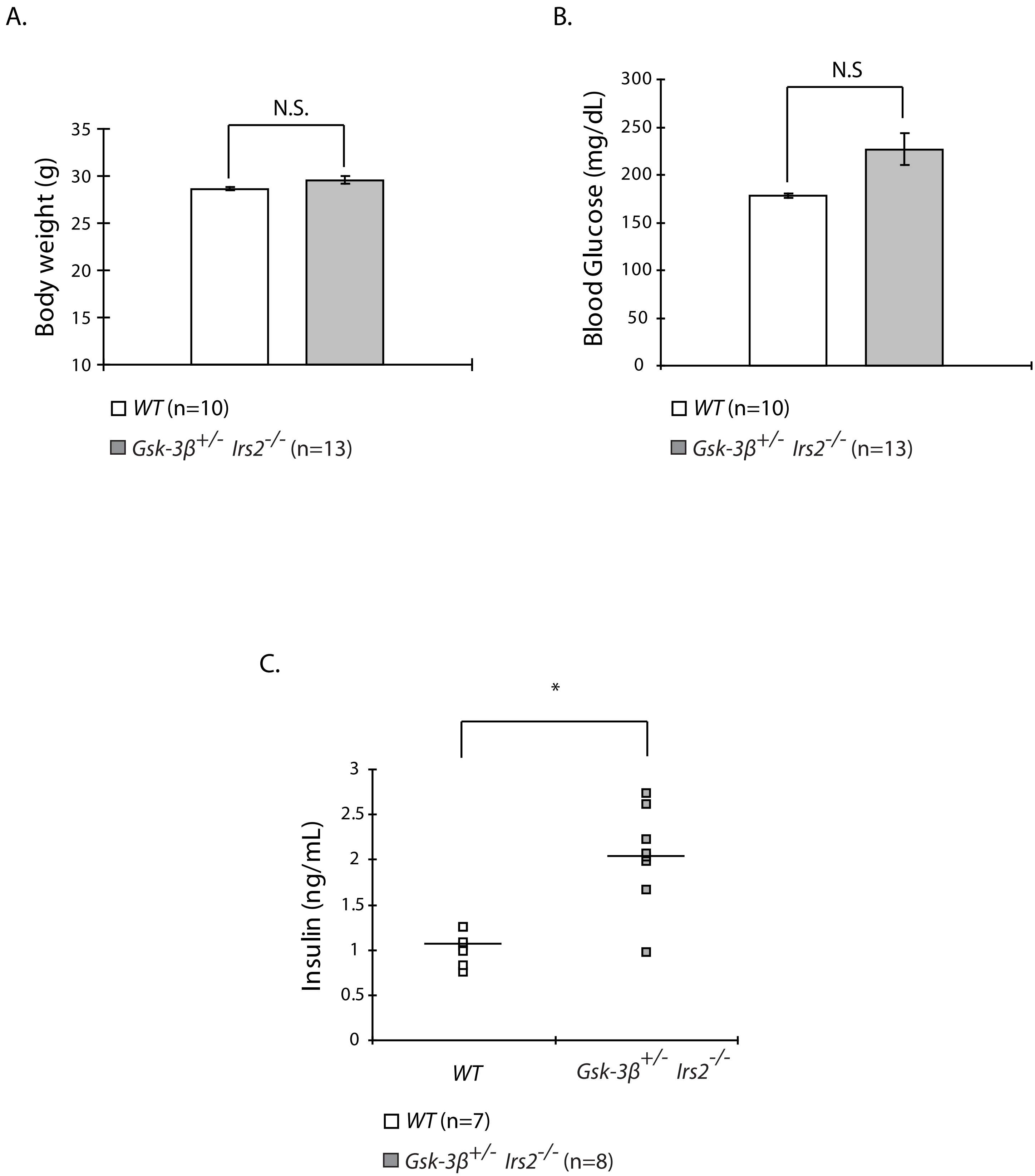
A.
N.S.
Body weight (g)
35
30
25
20
15
10
WT (n=10)
Gsk-3β+/- Irs2-/- (n=13)
B.
N.S
Blood Glucose (mg/dL)
300
250
200
150
100
50
0
WT (n=10)
Gsk-3β+/- Irs2-/- (n=13)
C.
*
Insulin (ng/mL)
3
2.5
2
1.5
1
0.5
0
WT
Gsk-3β+/- Irs2-/-
WT (n=7)
Gsk-3β+/- Irs2-/- (n=8)
