# Supplementary material for: Genetic Deficiency of Glycogen Synthase Kinase-3β Corrects Diabetes in Mouse Models of Insulin Resistance
Source: PLoS Biol. 2008 Feb 19;6(2):e37. doi: 10.1371/journal.pbio.0060037 (PMC2245985; doi:10.1371/journal.pbio.0060037)
Supplement: Supplementary file 3 [file pbio.0060037.sg003.pdf]

S3

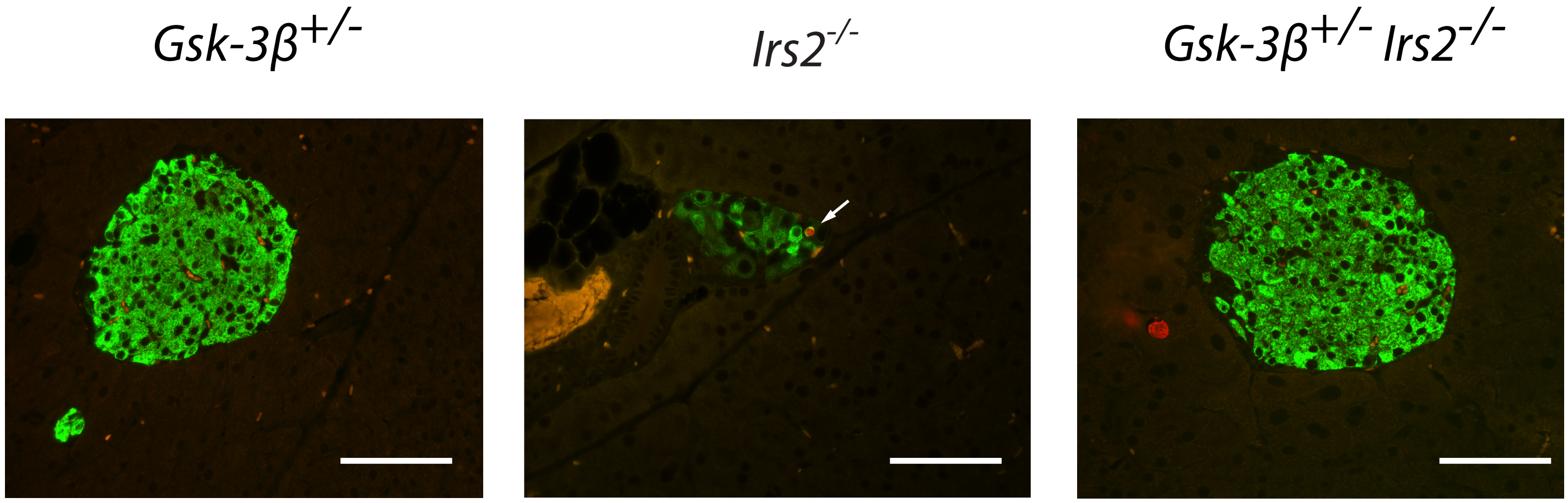

TUNEL, Insulin
