# Supplementary material for: Genetic Deficiency of Glycogen Synthase Kinase-3β Corrects Diabetes in Mouse Models of Insulin Resistance
Source: PLoS Biol. 2008 Feb 19;6(2):e37. doi: 10.1371/journal.pbio.0060037 (PMC2245985; doi:10.1371/journal.pbio.0060037)
Supplement: Supplementary file 4 [file pbio.0060037.st001.pdf]

Table S1

fasting blood glucose concentration of 6 and 8 week-old mice

Genotype	Blood Glucose (mg/dL) 6w	8w
Wild-type (n=10)	84(± 1.3)	87(± 2.2)
Gsk-3β$^{+/-}$ (n=9)	81(± 2.9)	75(± 1.9)
Irs2$^{-/-}$ (n=15)	127(± 1.9)***	148(± 1.9)***
Gsk-3β$^{+/-}$ Irs2$^{-/-}$ (n=12)	125(± 1.3)***	123(± 1.5)***

$^{***}p < 0.001$ by one-factor student t-test compared with Wild-type.
